# Evaluation and comparison of different machine learning approaches to auditory spectro-temporal receptive field estimation

**DOI:** 10.1186/1471-2202-12-S1-P4

**Published:** 2011-07-18

**Authors:** Arne F Meyer, Jan P Diepenbrock, Frank W Ohl, Jörn Anemüller

**Affiliations:** 1 Medical Physics, Department of Phyics, Carl-von-Ossietzky University, Oldenburg, Germany; 2 Leibniz Institute for Neurobiology and Institute of Biology, Otto-von-Guericke University, Magdeburg, Germany

## 

The linear spectro-temporal receptive field (STRF) is a well-known approach to describe which features are encoded by auditory cortical neurons [1]. It is defined as the linear filter that, when convolved with the envelope of a stimulus, gives a linear estimate of the spike rate evoked by that stimulus. A common STRF estimation method is reverse correlation, also known as spike-triggered average (STA), where the stimulus parts preceding the spikes are averaged in a specific time window. Linear regression approaches estimate an STRF based on the ensemble-averaged response spike rate. In [3] we have shown that the linear STRF model can be reformulated in terms of linear classification and a novel method using a support vector machine (SVM) classifier has been presented. Hence, given a set of stimuli with evoked response ensembles measured for a neuron the STRF of that specific neuron can be estimated using any of these methods.

Reverse correlation, linear regression and the SVM-based approach are evaluated using neural recordings from the primary auditory cortex of mongoelan gerbils [2] and synthetic data created using an inhomogeneous Linear-nonlinear Poisson (LNP) model with refractory period [4]. In the LNP model, STRFs with different characteristics are used in the linear stage of the model and the average spike rate and the number of trials are varied. Complexes of frequency modulated (FM) sweeps as described in [2] are used as stimuli for both types of data. All methods are tested and evaluated using 5-fold cross-validation (CV). Model complexity in terms of Principal Components (PC) for linear regression and the SVM classifier is determined by using the least complex model within one standard deviation of the maximum mean coherence between estimated and predicted spike rate. The resulting STRFs are evaluated using mean coherence, the area under the receiver operating curve (AUC) for single spike classification and STRF variability for the different CV folds.

**Figure 1:**
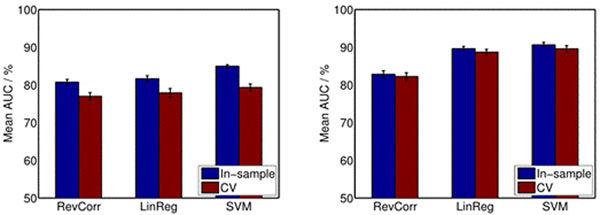
Area under the ROC curve for the different STRF estimation methods for 1 trial (left) and 10 trials (right). In-sample results are shown in blue and cross-validation results are shown in red. The datasets were created using a Linear-nonlinear Poisson (LNP) model.

As shown in Figure 1, linear regression and SVM classification produce STRFs that are better predictors for responses of cortical neurons than traditional reverse correlation. The classification-based approach yields the best response field characterization for single-trial data as well as for data with multiple trials. In general, linear regression and SVM classification produce STRFs with very similar structure and high predictive power for rather linear neurons validating the reformulation of the STRF model.
